# Supplementary material for: Mixed Methods Lot Quality Assurance Sampling: A novel, rapid methodology to inform equity focused maternal health programming in rural Rajasthan, India
Source: PLoS One. 2021 Apr 29;16(4):e0250154. doi: 10.1371/journal.pone.0250154 (PMC8084134; doi:10.1371/journal.pone.0250154)
Supplement: S1 File — (DOCX) [file pone.0250154.s001.docx]

**S1 File. LQAS parameters: Sample size, decision rule, coverage thresholds, and acceptable error.**

To design LQAS system, the user must specify upper and lower thresholds for each indicator. The upper threshold is often the “coverage target” (or in this study, the expected coverage of a program), and the lower threshold is an unacceptably low level of coverage. In addition to upper and lower thresholds, alpha (the maximum allowable probability classifying a “high” coverage area as “low”) and beta (the maximum allowable probability of classifying a “low” coverage area as “high”) errors are pre-specified parameters. The upper threshold, lower threshold, alpha- and beta-errors determine the minimum sample size needed and the corresponding decision rule (d) that marks the cut-off between “high” and “low” coverage areas. For each indicator of interest, the respondent is marked as giving a “positive” or “negative” response (i.e. “yes” or “no,” respectively, to the question “did you have a facility-based delivery?”). Positive responses for each supervision area are totaled and compared to the relevant decision rule (d). If the total number of positive responses is above the decision rule the supervision area is classified as “high,” otherwise it is classified as “low.”

The two major indicators for this study are: 1) ‘Full’ antenatal care coverage (defined according to NRHM as at least 3 ANC checkups, at least 1 tetanus vaccination, and 100+ iron folic acid tablets/syrup consumed); and 2) Facility-based delivery coverage (defined as having delivered in a health facility).

According to India’s Annual Health Survey conducted in 2012-13, full antenatal care coverage in rural Sri Ganganagar was 15.2%. Full ANC coverage was even lower for the lowest wealth quintile at 2.5% in the District Level Health Household and Facility Survey (DLHS-3) conducted in 2006, the last year disaggregated data is available. Assuming that the National Rural Health Mission (NRHM), implemented in 2005, has had a positive impact on ANC coverage, and keeping in mind that this project aims to focus on the most vulnerable women, we set a lower threshold of 10% for defining very low coverage. The upper threshold for ANC, which is designated as ‘adequate’ coverage given current conditions, was 40% and represents a 20-percentage point increase over the coverage of Rajasthani women in the wealthiest quintile (19.6%) in DLHS-3.

The Annual Health Survey, 2012-13 indicates a facility-based delivery coverage of 87.9% for rural Sri Ganganagar. Referring again to DLHS-3, we see a large range between different wealth quintiles (36.6% safe delivery for the poorest and 82.2% for the wealthiest). Using similar logic for the ANC indicator, we set the upper target coverage level at 85%. The lower threshold for this indicator was set at 55%. For acceptable error, we set both the alpha- and beta-errors to 10% for both indicators. These LQAS parameters are summarized in Table S1. The calculated sample sizes (m), decision rule (d) and actual errors associated with the LQAS system for each indicator are listed in table 2.3

| **Table S1. LQAS predetermined parameters: P-upper, P-lower, and alpha and beta errors.** | | | | |
| --- | --- | --- | --- | --- |
| **Indicator** | **P-upper (%)** | **P-lower (%)** | **alpha**  **(max)** | **beta**  **(max)** |
| Full ANC | 40 | 10 | ≤0.10 | ≤0.10 |
| FBD | 85 | 55 | ≤0.10 | ≤0.10 |

| **Table S2. LQAS parameters: calculated sample size (m) and decision rule (d).** | | | | |
| --- | --- | --- | --- | --- |
| **Indicator** | **m** | **d** | **alpha** | **beta** |
| Full ANC | 15 | 4 | 0.091 | 0.056 |
| FBD | 16 | 12 | 0.079 | 0.085 |

**Sample size (m) and decision rule (d) calculations (Stata output)**

*ANC coverage

clear

*User inputs pu and pl, survey specific

global pu 0.4

global pl 0.10

*User inputs alpha beta levels, survey-specific

global alpha_level 0.1

global beta_level 0.1

*** Do not edit. Calculates n and d using

global n 1

global test 0

while $test == 0{

global n = ($n +1)

clear

quietly set obs $n

generate d = _n

generate alpha = 1-binomialtail($n, d, $pu)

generate beta = binomialtail($n, d, $pl)

generate dummy = 1

quietly sum dummy if alpha <= $alpha_level & beta <= $beta_level

global test = r(N)

}

generate n = $n

drop dummy

order n d alpha beta

list n d alpha beta if alpha <= $alpha_level & beta <= $beta_level, noobs

+------------------------------+

| n d alpha beta |

|------------------------------|

| 15 4 .0905019 .0555556 |

+------------------------------+

.

end of do-file

*FBD coverage

clear

*User inputs pu and pl, survey specific

global pu 0.85

global pl 0.55

*User inputs alpha beta levels, survey-specific

global alpha_level 0.1

global beta_level 0.1

*** Do not edit. Calculates n and d using

global n 1

global test 0

while $test == 0{

global n = ($n +1)

clear

quietly set obs $n

generate d = _n

generate alpha = 1-binomialtail($n, d, $pu)

generate beta = binomialtail($n, d, $pl)

generate dummy = 1

quietly sum dummy if alpha <= $alpha_level & beta <= $beta_level

global test = r(N)

}

generate n = $n

drop dummy

order n d alpha beta

list n d alpha beta if alpha <= $alpha_level & beta <= $beta_level, noobs

+-------------------------------+

| n d alpha beta |

|-------------------------------|

| 16 12 .0790513 .0853092 |

+-------------------------------+

.

end of do-file
