# Supplementary material for: Mixed Methods Lot Quality Assurance Sampling: A novel, rapid methodology to inform equity focused maternal health programming in rural Rajasthan, India
Source: PLoS One. 2021 Apr 29;16(4):e0250154. doi: 10.1371/journal.pone.0250154 (PMC8084134; doi:10.1371/journal.pone.0250154)
Supplement: S2 File — (DOCX) [file pone.0250154.s002.docx]

**S2 File. LC-LQAS sample size calculation.**

Since both ANC and FBD indicators would be assessed at the same time, we will use a sample size (m) of 16 for each supervision area. Using procedures described in Hedt et al. (2008) and restricting confidence intervals to +/- 10%, the parameters in Table S3 are used to calculate the required number of randomly sampled panchayats. Given the current population estimates for women aged 18-49 in Sri Ganganagar in the 2011 Indian Census (Table S4), the number of randomly sampled clusters required to satisfy all conditions is n = 18. The total number of surveyed women required to be to satisfy all conditions is therefore (16 x 18) N = 288.

| **Table S3. Parameters for calculation of cluster sample size (n) using proportion of rural women aged 18-49 in Sri Ganganagar, Rajasthan, India.** | | |
| --- | --- | --- |
| Total number of supervision areas | N | 118 |
| Sample Size per SA | m | 16 |
| Total Female Population (18-49)^*^ | N*_cen_ | 93,836 |
| Average Square SA Population (18-49) | $\bar{M}$^2^ | 686,037 |
| Estimate of Intracluster Correlation^†^ | ρ | 0.10 |
| ^*^Available census data do not list population totals of females between the ages 18 and 49 per village. This population is derived from the proportion of rural females aged 18-49 (321,537) to the total female rural population (675,467): 321,537/675,467 = 0.4760.  ^†^The estimate of 0.10 was used, since no previous ICC was found.  Source: Government of India. Census of India, 2011. Available at: http://www.censusindia.gov.in/ | | |

$$\bar{M}^{2}=\frac{1}{N}\sum_{i=1}^{N} M_{i}^{2}=\frac{80,952,371}{118}= 686,037$$

$$n=N(1+\left( m-1 \right)\hat{\rho})\left[ \left( \frac{l_{max}N_{cen}^{*}}{1.96} \right)^{2}\left( \frac{(m-1)(1-\hat{\rho})}{N\bar{M^{2}}} \right)+m\hat{\rho} \right]^{-1}$$

$$n=118(1+\left( 16-1 \right)0.10)\left[ \left( \frac{0.2(93,836)}{1.96} \right)^{2}\left( \frac{(16-1)(1-0.10)}{118 (686,037)} \right)+16(0.10) \right]^{-1}$$

$n=295$ $\left[ 91,682,579.09\left( \frac{13.5}{80,952,366} \right)+1.6) \right]^{-1}$

$n=295$ $\left[ 15.29+1.6) \right]^{-1}$

$$n=295 \left( \frac{1}{16.89} \right)=17.47$$

$$n=18$$

Sample size = m x n = 16 x 18 = 288 individuals to be sampled

There are several assumptions made about the survey population. These include the assumption that the density of eligible, low-income women is the same as the density of non-pucca houses, that the proportion of poor houses is consistent across villages, that there is one eligible woman per household, and that the 2011 Indian Census is still representative. There is also an assumption that women from low-income families are indeed vulnerable. Undoubtedly, there are vulnerable women from wealthy households who would be excluded from our study.

We also assumed that the intracluster correlation coefficient (ICC), which reflects the extent to which variations in FBD and ANC coverage proportions are dependent on the cluster from which a respondent comes from, is ‘low,’ which in our case is defined as 0.10.

**Table S4. Census data for each *Panchayat* (Supervision Area), population and population squared (**$\bar{\boldsymbol{M}}$***^2^*).**

|  | Supervision Area (SA) *(Panchayat)* | Sub-district (*Tehsil*) | Female Population | Proportion of females (age 18-49) (female population x 0.4760)* | Female Population Squared (*Mi^2^*) (age 18-49) |
| --- | --- | --- | --- | --- | --- |
| 1 | 10 O | Karanpur | 1,485 | 707 | 499,651 |
| 2 | 12 H (MOHALA) | Karanpur | 1,155 | 550 | 302,258 |
| 3 | 13 FF (MANAKSAR) | Karanpur | 1,572 | 748 | 559,911 |
| 4 | 14 FF | Karanpur | 1,205 | 574 | 328,994 |
| 5 | 14 S (MAGHIWALA | Karanpur | 1,535 | 731 | 533,864 |
| 6 | 15 O | Karanpur | 1,339 | 637 | 406,233 |
| 7 | 2 F-C (MUKAN) | Karanpur | 1,187 | 565 | 319,239 |
| 8 | 2 FFA | Karanpur | 741 | 353 | 124,409 |
| 9 | 2 M (PHUSEWALA) | Karanpur | 1,525 | 726 | 526,931 |
| 10 | 2 T (MALKANA KALAN) | Karanpur | 2,243 | 1,068 | 1,139,915 |
| 11 | 2 W (GURUSAR) | Karanpur | 1,776 | 845 | 714,661 |
| 12 | 2 X | Karanpur | 1,148 | 546 | 298,605 |
| 13 | 25 F (GULABEYWALA) | Karanpur | 1,344 | 640 | 409,272 |
| 14 | 25 H (DALPATSINGHPURA) | Karanpur | 2,075 | 988 | 975,551 |
| 15 | 27 F (KAMEENPURA) | Karanpur | 1,398 | 665 | 442,821 |
| 16 | 3 O | Karanpur | 1,604 | 764 | 582,938 |
| 17 | 32 F (ARAYAN) | Karanpur | 2,677 | 1,274 | 1,623,718 |
| 18 | 36 H (NAGGI) | Karanpur | 1,749 | 833 | 693,096 |
| 19 | 4 S (MALKANA KHURD) | Karanpur | 1,827 | 870 | 756,295 |
| 20 | 42 H | Karanpur | 1,138 | 542 | 293,426 |
| 21 | 43 GG (KHARALA) | Karanpur | 1,460 | 695 | 482,969 |
| 22 | 46 F (MODA) | Karanpur | 1,758 | 837 | 700,248 |
| 23 | 48 GG (SHRINAGAR) | Karanpur | 1,641 | 781 | 610,142 |
| 24 | 50 F (ROOPNAGAR) | Karanpur | 1,511 | 719 | 517,300 |
| 25 | 52 GG (GULABEWALA) | Karanpur | 1,439 | 685 | 469,176 |
| 26 | 56 F | Karanpur | 1,405 | 669 | 447,267 |
| 27 | 6 FA (RADEWALA) | Karanpur | 1,736 | 826 | 682,831 |
| 28 | 61 F | Karanpur | 1,272 | 605 | 366,596 |
| 29 | 6V (NOOR) | Karanpur | 2,076 | 988 | 976,492 |
| 30 | 8 V | Karanpur | 1,146 | 545 | 297,566 |
| 31 | 9 FA (MAJHIWALA) | Karanpur | 1,524 | 725 | 526,240 |
| 32 | 9 FF (BAROPAL | Karanpur | 1,518 | 723 | 522,105 |
| 33 | GHARGAWALI | Karanpur | 1,367 | 651 | 423,400 |
| 34 | KEEKARWALI | Karanpur | 1,247 | 594 | 352,328 |
| 35 | TIBA MOTASAR KHOONI | Karanpur | 1,223 | 582 | 338,896 |
| 36 | 1 EEA-II (GHAMOODWALI) | Padampur | 2,134 | 1,016 | 1,031,817 |
| 37 | 1 PS | Padampur | 1,358 | 646 | 417,843 |
| 38 | 11 EEA (PHARSEWALA) | Padampur | 1,285 | 612 | 374,128 |
| 39 | 16 BB (04) | Padampur | 1,909 | 909 | 825,707 |
| 40 | 19 BB | Padampur | 2,297 | 1,093 | 1,195,462 |
| 41 | 20 BB | Padampur | 1,901 | 905 | 818,801 |
| 42 | 23 BB | Padampur | 1,958 | 932 | 868,639 |
| 43 | 3 EEA (PHARSEWALA) | Padampur | 1,661 | 791 | 625,105 |
| 44 | 3 RB-A(18) | Padampur | 1,259 | 599 | 359,141 |
| 45 | 31 RB (PHAKEERWALI)(20) | Padampur | 1,945 | 926 | 857,143 |
| 46 | 34 LNP | Padampur | 2,949 | 1,404 | 1,970,441 |
| 47 | 35 BB | Padampur | 1,769 | 842 | 709,038 |
| 48 | 37 GG (GHUDHDUWALA) | Padampur | 1,146 | 545 | 297,566 |
| 49 | 39 RB | Padampur | 943 | 449 | 201,482 |
| 50 | 4 BB | Padampur | 1,191 | 567 | 321,394 |
| 51 | 4 DD (DELWAN) | Padampur | 1,178 | 561 | 314,416 |
| 52 | 4 EE (SAWANTSAR) | Padampur | 2,192 | 1,043 | 1,088,667 |
| 53 | 4 JJ | Padampur | 1,642 | 782 | 610,886 |
| 54 | 4 NN (CHANANA)(05) | Padampur | 1,129 | 537 | 288,803 |
| 55 | 46 RB | Padampur | 1,413 | 673 | 452,375 |
| 56 | 5 BBA | Padampur | 1,652 | 786 | 618,349 |
| 57 | 5 KK | Padampur | 1,759 | 837 | 701,044 |
| 58 | 51 RB (TAMKOT) | Padampur | 1,653 | 787 | 619,098 |
| 59 | 52 LNP (MANJHUWAS) | Padampur | 1,326 | 631 | 398,383 |
| 60 | 54 LNP | Padampur | 1,188 | 565 | 319,777 |
| 61 | 6 RB (JALAUN) | Padampur | 2,091 | 995 | 990,654 |
| 62 | 60 LNP (RIDMALSAR) | Padampur | 2,522 | 1,200 | 1,441,133 |
| 63 | 69 LNP | Padampur | 1,959 | 932 | 869,526 |
| 64 | 7 DD | Padampur | 1,241 | 591 | 348,945 |
| 65 | 75 LNP (RATANPURA) | Padampur | 2,144 | 1,021 | 1,041,510 |
| 66 | 8 NN | Padampur | 1,185 | 564 | 318,164 |
| 67 | 83 LNP (JODKIYA) | Padampur | 1,512 | 720 | 517,985 |
| 68 | 9 BB (RATEWALA) | Padampur | 2,177 | 1,036 | 1,073,818 |
| 69 | BINGH BAYALA | Padampur | 3,261 | 1,552 | 2,409,437 |
| 70 | JIWANDESAR | Padampur | 2,638 | 1,256 | 1,576,752 |
| 71 | NARSINGHPURA BARANI | Padampur | 2,250 | 1,071 | 1,147,041 |
| 72 | 1 MK-B (UDSAR) | Raisinghnagar | 2,408 | 1,146 | 1,313,793 |
| 73 | 1 NZP-A (BAJUWALA) | Raisinghnagar | 1,959 | 932 | 869,526 |
| 74 | 10 TK | Raisinghnagar | 1,183 | 563 | 317,091 |
| 75 | 11 TK | Raisinghnagar | 1,511 | 719 | 517,300 |
| 76 | 12 NRD (BISHANPURA SIGD | Raisinghnagar | 2,537 | 1,208 | 1,458,327 |
| 77 | 13 SAD (SATJANDA) | Raisinghnagar | 1,664 | 792 | 627,365 |
| 78 | 13 TK (JAGATSINGHWALA) | Raisinghnagar | 1,925 | 916 | 839,606 |
| 79 | 15 PTD-A (SAMEJA) | Raisinghnagar | 2,552 | 1,215 | 1,475,622 |
| 80 | 16 PS | Raisinghnagar | 1,333 | 635 | 402,600 |
| 81 | 17 TK | Raisinghnagar | 1,467 | 698 | 487,612 |
| 82 | 2 IWM (SARDARPURABIKA) | Raisinghnagar | 1,637 | 779 | 607,171 |
| 83 | 21/23 NP (THANDI) | Raisinghnagar | 1,829 | 871 | 757,951 |
| 84 | 22 PS | Raisinghnagar | 1,642 | 782 | 610,886 |
| 85 | 22 PTD-B | Raisinghnagar | 1,711 | 814 | 663,306 |
| 86 | 22/23 F (LUHARA) | Raisinghnagar | 1,142 | 544 | 295,492 |
| 87 | 23 RB (SANGRANA) | Raisinghnagar | 2,180 | 1,038 | 1,076,780 |
| 88 | 27 PS (LIKHMEWALA) | Raisinghnagar | 2,824 | 1,344 | 1,806,938 |
| 89 | 3 FFB (THADEWALA) | Raisinghnagar | 1,187 | 565 | 319,239 |
| 90 | 3 MK | Raisinghnagar | 1,473 | 701 | 491,609 |
| 91 | 30 PS-A (BISHANPURA) | Raisinghnagar | 858 | 408 | 166,797 |
| 92 | 32 PS-A (SANWANTS | Raisinghnagar | 1,169 | 556 | 309,630 |
| 93 | 36 NP (NANUWALA) | Raisinghnagar | 1,648 | 784 | 615,359 |
| 94 | 4 FFB (KANWARPUR | Raisinghnagar | 1,222 | 582 | 338,342 |
| 95 | 4 SAD (MALSAR) | Raisinghnagar | 1,999 | 952 | 905,398 |
| 96 | 43 PS | Raisinghnagar | 1,550 | 738 | 544,349 |
| 97 | 48 NP (BAGICHA) | Raisinghnagar | 1,863 | 887 | 786,393 |
| 98 | 5 NP (DABALA KHURD) | Raisinghnagar | 2,712 | 1,291 | 1,666,454 |
| 99 | 5 T K | Raisinghnagar | 868 | 413 | 170,708 |
| 100 | 56 RB (GANGUWALA | Raisinghnagar | 1,181 | 562 | 316,019 |
| 101 | 59 NP (SHAYAMGAR | Raisinghnagar | 1,582 | 753 | 567,057 |
| 102 | 6 FD (KHYALIWALA | Raisinghnagar | 1,690 | 804 | 647,124 |
| 103 | 6 JKM | Raisinghnagar | 1,853 | 882 | 777,973 |
| 104 | 60 RB (KIKARWALI) | Raisinghnagar | 1,597 | 760 | 577,861 |
| 105 | 66 RB | Raisinghnagar | 2,289 | 1,090 | 1,187,150 |
| 106 | 68 NP | Raisinghnagar | 1,794 | 854 | 729,220 |
| 107 | 7 PS | Raisinghnagar | 1,284 | 611 | 373,546 |
| 108 | 71 RB | Raisinghnagar | 1,991 | 948 | 898,166 |
| 109 | 75 NP | Raisinghnagar | 2,466 | 1,174 | 1,377,844 |
| 110 | 78 RB | Raisinghnagar | 1,012 | 482 | 232,046 |
| 111 | 79 RB-B (BHADAWAWALA) | Raisinghnagar | 1,188 | 565 | 319,777 |
| 112 | 8 LPM-A | Raisinghnagar | 1,733 | 825 | 680,473 |
| 113 | 84 RB-B | Raisinghnagar | 1,709 | 813 | 661,756 |
| 114 | 9/10 KSD (MOHKAMWALA) | Raisinghnagar | 2,482 | 1,181 | 1,395,782 |
| 115 | BHOMPURA | Raisinghnagar | 1,745 | 831 | 689,930 |
| 116 | KARARWALI | Raisinghnagar | 1,288 | 613 | 375,877 |
| 117 | KHATA | Raisinghnagar | 1,042 | 496 | 246,008 |
| 118 | THAKARI | Raisinghnagar | 2,293 | 1,091 | 1,191,302 |
|  |  |  | **197,135** | **93,836** | 80,952,371 |
|  |  |  |  |  | $\bar{M}$*^2^* =686,037 |
| *Available census data do not list population totals of females between the ages 18 and 49 per village. This population is derived from the proportion of rural females aged 18-49 (321,537) to the total female rural population (675,467): 321,537/675,467 = 0.4760. | | | | | |
